# Meta review of systematic and meta analytic reviews on movement differences, effect of movement based interventions, and the underlying neural mechanisms in autism spectrum disorder

**DOI:** 10.3389/fnint.2013.00016

**Published:** 2013-03-26

**Authors:** Motohide Miyahara

**Affiliations:** Movement Development Clinic, School of Physical Education, University of OtagoDunedin, New Zealand

**Keywords:** autism, movement, developmental coordination disorder, motor development, MRI and fMRI

## Abstract

**Purposes:** To identify and appraise evidence from published systematic and meta analytic reviews on (1) movement differences of individuals with autism spectrum disorders (ASD); (2) the effects of movement based interventions for ASD; (3) hypothesized underlying neural mechanisms for the movement characteristics.

**Methods:** A meta review of published systematic and meta analytic reviews on movement differences, structural, and functional brain anomalies in ASD and the effects of movement based interventions for individuals with ASD between 1806 and October 2012. The methodological quality of the identified systematic and meta analytic reviews was independently assessed by two assessors with the assessment of multiple systematic reviews (AMSTAR).

**Results:** The search yielded a total of 12 reviews that met the inclusion/exclusion criteria. The methodological quality of the reviews varied, but the review conclusions were similar. Although individuals with ASD generally perform less well than age-matched controls in developmental movement tasks, there are few exceptions whose movement abilities are intact. Most movement based interventions report their efficacies. However, all existing studies employ the research design that is inherently incapable of providing strong evidence, and they often fail to report the extent of psychosocial interactions within the movement interventions. The hypothesized neural mechanisms are still under development and speculative in nature.

**Conclusions:** It is premature to designate movement disturbance as a core symptom of ASD. The effects of movement based interventions on the present ASD core symptoms need to be further validated by stronger evidence and verified theoretical mechanisms linking ASD with movement disorders.

## Introduction

The core symptoms of autism spectrum disorders (ASD) encompass impairments in social interaction and communication, as well as circumscribed interest and fixed behaviors (American Psychiatric Association, 2012). A cross-cutting dimension of these clinical signs is the development of movement skills that are observable, functional, goal-oriented, and acquired as a result of practice. For example, the movements of the vocal cord and the other body parts are essential for communicative speech, gesture, writing, and typing. Stereotypies and preoccupation can be only inferred from observed physical movement patterns. Thus, movement disturbances seem to co-exist with ASD, but the relations between the two disorders have not been critically appraised or adequately synthesized from the previous reviews.

In this meta review, I will evaluate the methodological quality of reviews, re-examine the strength of evidence, the homogeneity of studies and research designs, and integrate the findings to answer my three research questions: movement differences, effect of movement based interventions, and the underlying neuronal mechanisms that link ASD with movement disturbance.

## Methods

My search was based on the electronic databases available at the University of Otago, Dunedin, New Zealand: Web of Knowledge (1898–2012), Medline (1950–2012), PsychINFO (1806–2012), EMBASE (1980–2012), and ERIC (1987–2012) on June 12, 2012. I used the combinations of three or four keywords from the following inclusion criteria: (1) type of paper: systematic review or meta analysis; (2) population or comparison: autism, pervasive developmental disorders, autism spectrum disorder, Asperger disorder; (3) intervention: sport, exercise, physical exercise, physical activity; (4) primary outcome: movement skills, motor skills, motor coordination, physical fitness, imitation; secondary outcome: social skills, autistic behaviors, self-perception, and academics; (5) magnetic resonance imaging (MRI). I included only published studies in English, and excluded “gray” and “figitive” literature. To conduct duplicate study selection, a reference librarian served as a reviews search coordinator. Any inconsistencies in our results were resolved through subsequent collaborative search, reading of respective journal articles, and discussion.

The content of identified review studies was assessed with respect to the professional field of reviewers, type and topic of reviews, numbers of reviewed studies, year range of the studies, total number of subjects, level of evidence, review method, outcome variables, and theoretical interpretation. The methodological quality of the reviews was assessed by two independent assessors with the assessment of multiple systematic review (AMSTAR) tool (Shea et al., 2007). Disagreements were resolved by discussion.

## Results

Our search yielded a total of 12 studies. As shown in Table 1, four studies examined the existing evidence of movement differences, the other four evaluated the evidence of movement based treatment effects, and the remaining four studies synthesized the foregoing studies that investigated the neuronal mechanisms that link ASD and movement disturbance. I excluded three studies (Redcay and Courchesne, 2005; Sugranyes et al., 2011; Ipser et al., 2012) from further analysis because they did not address my research questions directly. The AMSTAR methodological quality scores of the 12 reviewed studies ranged from 20 (Emck et al., 2009) to 67% (Petrus et al., 2008; Stanfield et al., 2008) of the relevant criteria (Table 2).

**Table 1:** **Characteristics of reviews which met the inclusion criteria (*N* = 12)**.

**First author (Year)**	**Professional field of the reviewers**	**Type of review**	**Topic of review**	**No. of reviewed studies**	**Year range of the studies**	**Total no. of subjects**	**Levels of reviewed studies**	**Method used in reviewed studies**	**Outcome variables**	**Theoretical interpretations offered**
Williams (2004)	Pediatrics, Psychology	Systematic	Imitation difference	21	1966–2002	281	Not assessed	Comparative	Action imitation	Yes
Emck (2009)	Kinesiology, Psychiatry	Systematic	Movement difference	5^*^	1997–2007	Not reported	Not assessed	Comparative	Gross motor performance, self-perception	Yes
Fournier (2010)	Kinesiology	Meta analytic	Movement difference	51	1980–2009	Not reported	Not assessed	Comparative	Motor coordination	Yes
Downey (2012)	Physicla Therapy	Systematic	Movement difference	49	Not reported	Not reported	Level 2–3	Comparative	Motor development, imitation, posture, dyspraxia	Yes
Baranek (2002)	Education	Systematic	Treatment effect	4^∧^	1980–1993	Not reported	Level 3–4	Case series	Sensory motor functions, behavior	Yes
Petrus (2008)	Physicla Therapy	Systematic	Treatment effect	7	1982–2003	25	Level 2–5	Case series	Stereotypic behaviors	No
Lang (2010)	Education	Systematic	Treatment effect	18	1974–2007	64	Not assessed	Case series	Behavior, academics, physical fitness	Yes
Sowa (2012)	Neuroscience	Meta analytic	Treatment effect	16	1991–2011	133	Not assessed	Case series	Motor skills, social skills	No
Stanfield (2008)	Psychiatry	Meta analytic	Structural difference	46	1984–2006	Over 1600	Not assessed	Comparative	Brain volume	Yes
Müller (2011)	Psychology, Neuroscience	Systematic	Connectivity difference	32	2004–2010	Not reported	Not assessed	Comparative	Functional connectivity	Yes
Philip (2012)	Psychiatry	Meta analytic	Brain activation difference	3^†^	1984–2009	24	Not assessed	Comparative	Brain activation	Yes
Nickl-Jockschat (2012)	Psychiatry, Neuroscience	Meta analytic	Structural difference	16	1999–2009	580	Not assessed	Comparative	Structural changes	Yes

Note: All Meta analytic reviews included systematic reviews.

* No. of movement difference studies only;

∧ No. of movement intervention studies only;

† No. of movement task studies only.

**Table 2:** **Methodological quality evaluated by the assessment of multiple systematic reviews tool (AMSTAR) (Shea et al., 2007)**.

**First author (Year)**	**1. Was an “a priori” design provided?**	**2. Was there duplicate study selection and data extraction?**	**3. Was a comprehensive literature search performed?**	**4. Was the status of publication (i.e., gray literature) used as an inclusion criterion?**	**5. Was a list of studies (included and excluded) provided?**	**6. Were characteristics of included studies provided?**	**7. Was the scientific quality of the included studies assessed and documented?**	**8. Was the scientific quality of the included studies used appropriately in formulating conclusions?**	**9. Were the methods used to combine the findings of studies appropriate?**	**10. Was the likelihood of publication bias assessed?**	**11. Were potential conflicts of interest included?**	**Total score**	**Percent**
Williams(2004)	CA	CA	Yes	CA	No	Yes	Yes	Yes	NA	NA	No	4/9	44
Emck (2009)	CA	CA	CA	CA	No	Yes	Yes	No	NA	No	No	2/10	20
Fournier (2010)	CA	Yes	Yes	Yes	Yes	Yes	Yes	No	No	Yes	No	7/11	64
Downey (2012)	CA	CA	No	No	No	Yes	Yes	No	NA	NA	No	2/9	22
Baranek (2002)	CA	No	Yes	CA	No	Yes	Yes	Yes	NA	NA	No	4/9	44
Petrus (2008)	CA	Yes	Yes	Yes	No	Yes	Yes	Yes	NA	NA	No	6/9	67
Lang (2010)	CA	Yes	Yes	No	No	Yes	Yes	Yes	NA	NA	No	5/9	56
Sowa (2012)	CA	CA	No	CA	No	Yes	No	No	No	Yes	No	2/11	18
Stanfield (2008)	CA	Yes	Yes	Yes	No	Yes	NA	NA	Yes	Yes	No	6/9	67
Müller (2011)	CA	Yes	No	No	No	Yes	NA	NA	Yes	No	No	3/9	33
Philip (2012)	CA	CA	Yes	Yes	No	Yes	NA	NA	Yes	No	No	4/9	44
Nickl-Jockschat (2012)	CA	CA	CA	No	No	Yes	NA	NA	Yes	No	No	2/9	22

Note: CA, can't answer; NA, not applicable.

## Movement differences of ASD

Of the four objective reviews available on this topic, Fournier et al.'s study (2010) is the only standard meta analytic review on movement differences between individuals with ASD and controls. The remaining three systematic reviews consist of Emck et al.'s (2009) examination of gross motor performance and self-perceived motor competence in ASD, Downey and Rapport's (2012) examination of the movement differences that limit motor activity, and Williams et al.'s (2004) investigation into the differential imitation ability in which they attempted to quantify the movement differences based on *p*-values. Thus, the four reviews analyzed different yet partially overlapping aspects of movements with each distinctive method.

Fournier et al. (2010) provide the only standard meta analysis that examines motor difference in ASD. As evident on the relatively high AMSTAR score (Table 2), their methodological procedures are fairly meticulous. They found a large effect (*ES* = 1.20) which they called *motor coordination deficit* in ASD. Upon scrutiny, however, the variables included in the meta analysis were not only standard assessment of motor coordination, but also sensory motor measures, the measures of letter height (Beversdorf et al., 2001), neurological soft signs (Tani et al., 2006), and even an indicator of the cognitive executive function (Coldren and Halloran, 2003).

Inheritant to meta analysis is the “apples and oranges” problem in that data of different nature are analyzed together, and this criticism may be overruled if the analysis aims to generalize to a higher-order class as “fruit” (Matt and Cook, 2009). Though Fournier et al. name the higher-order class of meta analysed dependent variables “motor coordination,” specific sensory motor measures are not usually considered as the indicators of motor coordination in assessing developmental coordination disorder (DCD). Because the proposed DSM-5 (American Psychiatric Association, 2012) allows dual diagnoses of ASD and DCD, it is more meaningful and clinically practical to exclusively use the standard assessment of motor coordination. Fournier et al.'s moderator variable analyses were limited to subtypes of ASD, without including the types of movement measures as a moderator variable. Hence, I computed a random effects model meta analysis on the six comparisons in the five studies that had used standard motor coordination measures. The aggregated standardized mean difference effect was significant, 2.91 (*SE* = 0.581; *p* < 0.001; *Z* = 5.01; *I*^2^ = 93.48; 95% *CI* = 1.774−4.051), larger than 1.20, the effect size of the all 51 studies, suggesting a high degree of comorbidity of ASD with DCD.

Emck et al.'s (2009) systematic review only covered gross motor performance and perceived competence. The authors concluded that children with ASD were clearly impaired in gross motor development. It is noteworthy that they acknowledge the existence of children with ASD whose gross motor performance fell within the normal range. Because this review neither evaluated the strength of evidence nor attempted quantitative synthesis, their conclusions need to be interpreted with caution.

Downey and Rapport (2012) categorized the movement differences into early motor development, gesture and motor imitation, postural control, and dyspraxia. The strategy for their literature search was described clearly, but the strength of evidence was not evaluated in this systematic review. The movement differences were summed up as *activity limitations* (World Health Organization, 2001) which should take both biological and social aspects into account. However, the authors focused on individual functional adaptation, and recommended physical therapists to promote functional intervention without adequately addressing the needs for social and environmental accommodation. It is open to question whether all aspects of movement differences actually reflect the limitations in motor activity.

Williams et al. (2004) conducted a review on the difference in action imitation between individuals with ASD and matched controls. Although the levels of evidence were not specifically evaluated, the authors ensured the quality of reviewed studies by only including those studies that had employed control groups into their review. Out of thus selected 21 studies, 17 studies were pooled into a meta analysis. The combined logit *p*-value <0.0005 indicated a significant group difference.

In summary, a meta review of the four review studies indicates substantial movement differences between young people with ASD and their typically developing counterparts through a limited quality and quantity of data synthesis. However, not all individuals with ASD have DCD (Emck et al., 2009). Therefore, movement disturbance cannot constitute a *core* symptom of ASD. If present, a comorbid diagnosis of DCD should be given in accordance with the proposed DSM-5 (American Psychiatric Association, 2012).

## Effects of movement based interventions

Four studies (Baranek, 2002; Petrus et al., 2008; Lang et al., 2010; Sowa and Meulenbroek, 2012) examined the effects of movement based interventions in individuals with ASD. Only Sowa and Meulenbroek (2012) conducted a meta analysis, and the other studies reviewed foregoing studies systematically. All four studies included the effects of physical exercise on ASD symptoms and movement functions.

In addition to the effects of physical exercise, which will be elaborated later with more recent reviews, Baranek (2002) covered sensory- and motor-based interventions, such as sensory integration therapy and sensory stimulation techniques, which have decreased in popularity recently. Although slightly outdated, her review is comprehensive for the covered range of movement based interventions. She also pointed out the issue of internal validity, particularly the degree of psychosocial interaction that occurred during physical exercise. This information is crucial to determine whether physical exercise alone or a combination of exercise and interpersonal interaction altered dependent variables. She maintained that the effect of physical exercise would be specific to the context of physical exercise, and it would not be generalized or transferred to social play.

Petrus et al.'s (2008) systematic review on exercise intervention effects targeted the outcome variable of stereotypic behaviors in children with ASD. They reviewed seven studies which used either the case series or case study design with small sample sizes (*N* < 6). Petrus et al. classified the studies by Kern et al. (1982) and Kern et al. (1984) as Level II: smaller RCT. However, these studies had no control group, and therefore, should be reclassified as Level IV: case series. Given the weak evidence of the reviewed studies, Petrus et al.'s claim of “weak to moderately strong evidence” (p. 142) should be revised to *weak* evidence.

Lang et al.'s (2010) systematic review overlaps with Petrus et al.'s (2008) review, but included wider outcome variables other than stereotypic behaviors. As in the case of Petrus et al.'s (2008) review, the appraised studies were characterized by small sample sizes (*N* < 9) and the use of time-series analysis to evaluate the intervention outcome (7 of the 18 studies). This review provided critical information that 15 of the 18 studies had involved teaching exercise, mostly jogging, to individuals with ASD by modeling, physical guidance, verbal reinforcement and contingency management. One study even used jogging in social plays, such as follow-the-leader and tag. Such a psychosocial component in the “physical” intervention could explain the improvement in the behavioral and academic domains, as well as in the physical domain. The authors narratively evaluated the research methodologies and the intervention outcomes, but neither categorical classification of evidence levels nor meta analysis was performed. Yet Lang et al. (2010) acknowledged the limitations of reviewed research being the fact that no research employed the experimental design, but depended on time-series analysis. An advantage of time-series analysis lies in its capability to infer the effect of intervention on an individual without considering inter-individual differences. The inevitable corollary of the advantage reduces generalizability. Hence, the reported benefits of physical exercises from the time-series data need to be confirmed by randomized control trials.

Sowa and Meulenbroek (2012) searched movement based intervention studies published between 1991 and 2011 with respect to the effects of the interventions on the physical and psychosocial domains in people with ASD. Of the 16 studies they identified, seven studies conducted individual physical exercise programs and nine studies administered group programs. All activity programs yielded significant progress on the assessed measures, but the individual programmes elicited significantly more improvement than the group interventions not only in the movement domain, but also in the social domain. A question may be raised as to whether physical exercise indirectly triggered the improvement in social functions through a yet unknown mechanism, or the psychosocial interactions that occurred during the physical exercise programs directly enhanced the social skills. Unfortunately, a majority of the original studies cited in the review failed to report the extent of psychosocial interactions in the “physical” exercises, making it difficult to determine the cause of the improvement in the social domain.

As the analytic method of the improvement rates indicates, Sowa and Meulenbroek's analysis examines only the differences before and after the interventions without considering the control groups. Indeed, this is still a meta analysis in the sense of aggregating studies, such an analytic method falls in Grade III-3 to IV level of evidence, which is regarded as either satisfactory or poor (NHMRC, 2000). The absence of the control groups in the analysis does not allow us to tease out the intervention effects from confounding factors, such as Hawthorne effect. No matter how large the combined sample size may be, no definite conclusion can be drawn from Sowa and Meulenbroek's meta analysis with regard to the causal effects of the individual or group motor interventions on the motor or the psychosocial domain. On an additional note, the authors seem to be unfamiliar with the difficulties that individuals with ASD face while engaging in complex team sports in which the environment changes constantly, as the authors wonder why there is no “naturalistic group-based sport intervention like soccer” (p. 56).

Thus, all four reviews reported the benefit of movement based interventions, physical exercise, in particular, on the physical and psychosocial domains. On closer scrutiny however, the extent of psychosocial intervention within the movement based interventions has not been quantified or partialed out, but confounded. Coupled with the limited generalizability of time-series analysis employed by a majority of the reviewed studies, the reported effects need to be interpreted cautiously with understanding of these limitations.

## Theoretical models linking movement disorders and ASD

Six out of the eight reviews on motor differences or motor interventions offered theoretical insights into the relations between ASD and motor functions, ranging from causal directions between the motor and the social domains to underlying neural structures and functions (Table 1).

In their systematic review, Downey and Rapport (2012) raised a question whether limited social behaviors of individuals with ASD prevented them from learning motor skills, or poor motor functions impoverished social life. This is a curious question, but it is extremely difficult to establish clear-cut causalities for methodological reasons.

Four review studies speculated the underlying mechanism between movement and autistic disorders. On the functional level, Williams et al. (2004) attributed the movement difference to an ASD specific deficit in self-other mapping ability particularly for the imitation tasks that have low congruence between semantic and visuomotor couplings. On the physiological level, Baranek (2002) cited Kern et al. (1982) and Kern et al. (1984) to explain the benefit of physical exercise in term of physiological responses, such as the secretion of neurotransmitters, beta-endorphins, and acetylcholine. Emck et al. (2009) attributed the co-occurrence of the multiple impairments to “an abnormal connectivity of brain system” (p. 512) based on functional correlations between motor, cognitive, and socio-emotional impairments. More specifically, Fournier et al. (2010) attribute motor dysfunctions to abnormalities in fronto-striatal connections and basal ganglia, based on the foregoing neurophysiological studies. Four reviews on actual neurophysiological studies provide us with the “state of art” hindsight of structural and functional MRI studies.

Stanfield et al. (2008) meta analyzed 46 volumetric MRI studies on regional brain size in ASD by adjusting age and IQ. The cerebrum and the cerebellum (vermal lobules VI-VII and VIII-X) of the ASD were larger than the control, whereas the corpus callosum was smaller in size. The authors related the enlarged cerebellum and its presumably disorganized connection with the cerebral regions to motor dysfunction, as well as cognitive and socio-emotional dysfunctions in ASD. Thus, the authors ascribed the link between movement disturbance and ASD to morphological changes in the brain.

In their systematic review and meta analysis of functional MRI research on ASD, Philip et al. (2012) identified three studies which had employed motor tasks of button pressing in MRI scanners. Compared to controls, individuals with ASD showed significantly different activation patterns (either hyper- or hypo-activation) in the motor regions (e.g., the cerebellum, the precentral gyrus, the basal ganglia) and attentional systems (the basal ganglia, the superior and inferior parietal lobules). The authors related the ASD groups' hyper-activation in the right inferior frontal gyrus and the hypo-activation in the left inferior parietal lobule to the *mirror neuron system hypothesis* in that these regions were involved in observation and execution of model movements. Note that Philip et al. were conservative in that they made no link between the differential brain activation patterns and the movement difference in ASD or the effect of movement based intervention on ASD.

Müller et al. (2011) reviewed 32 functional connectivity MRI studies of ASD, and found 22 studies supported the general underconnectivity hypothesis, whereas 11 studies did not support the hypothesis. The authors recognized the diversity in data analysis, suggesting that the discrepant findings might depend on each study's methodology. They interpreted underconnectivity as decreased efficiency of network interactions, and the increased functional connectivity as a malformation in experience-driven network. Müller et al. (2011) believed that all results represented the anomaly in white matter development which resulted in ASD symptomatology encompassing social, communicative, and movement disorders.

Nickl-Jockschat et al. (2012) meta analyzed 16 morphometric MRI studies and linked disturbances in the left pericental region, the left putamen, the right caudate, and the right parietal operculum with sensorymotor impairment in ASD.

The theoretical links between movement disorder and ASD have been explored in terms of the directions of influence and the neurophysiological levels. It is difficult to establish the direction of causalities. None of the neurophysiological evidence sufficiently accounts for the movement differences in ASD or why movement based interventions result in the improvement in the motor and psychosocial domains in ASD.

## Conclusive remarks

Accumulated studies indicate significant movement differences between ASD and control groups. However, the existence of individuals with ASD, who are free from movement problems, does not warrant the designation of movement disturbance as a core symptom of ASD. There is moderate to low quality evidence for the effects of movement based intervention on the motor, behavior, and psychosocial domains. Coupled with the limited descriptions on the psychosocial interactions during the movement based interventions, future research on the process and the outcome of movement based intervention needs to control and examine the interactions more precisely. Neurophysiological accounts for the movement differences and the effects of movement based intervention range from the size of brain regions, differential brain activation patterns during motor tasks, and functional connectivity. While none of these theoretical hypotheses can directly explain the movement differences or the effects of movement based interventions, they serve as useful theoretical models to be refined and tested in further research.

### Conflict of interest statement

The author declares that the research was conducted in the absence of any commercial or financial relationships that could be construed as a potential conflict of interest.
